# Pattern Matching for DNA Sequencing Data Using Multiple Bloom Filters

**DOI:** 10.1155/2019/7074387

**Published:** 2019-04-14

**Authors:** Maleeha Najam, Raihan Ur Rasool, Hafiz Farooq Ahmad, Usman Ashraf, Asad Waqar Malik

**Affiliations:** ^1^Fatima Jinnah Women University, Rawalpindi, Pakistan; ^2^Victoria University, Melbourne, Australia; ^3^College of Computer Sciences and Information Technology (CCSIT), King Faisal University, Alahsa 31982, Saudi Arabia; ^4^School of Electrical Engineering and Computer Science, National University of Sciences and Technology (NUST), Islamabad, Pakistan; ^5^Department of Information Systems, Faculty of Computer Science and Information Technology, University of Malaya, Kuala Lumpur, Malaysia

## Abstract

Storing and processing of large DNA sequences has always been a major problem due to increasing volume of DNA sequence data. However, a number of solutions have been proposed but they require significant computation and memory. Therefore, an efficient storage and pattern matching solution is required for DNA sequencing data. Bloom filters (BFs) represent an efficient data structure, which is mostly used in the domain of bioinformatics for classification of DNA sequences. In this paper, we explore more dimensions where BFs can be used other than classification. A proposed solution is based on Multiple Bloom Filters (MBFs) that finds all the locations and number of repetitions of the specified pattern inside a DNA sequence. Both of these factors are extremely important in determining the type and intensity of any disease. This paper serves as a first effort towards optimizing the search for location and frequency of substrings in DNA sequences using MBFs. We expect that further optimizations in the proposed solution can bring remarkable results as this paper presents a proof of concept implementation for a given set of data using proposed MBFs technique. Performance evaluation shows improved accuracy and time efficiency of the proposed approach.

## 1. Introduction

DNA sequencing is paving the way in practical applications including life sciences and agricultural fields. However, massive size of DNA sequence data sets available today poses significant computational challenges. Several algorithms have been proposed over the years to address these issues. The compression-based algorithms have gained the attention of researchers recently due to their efficient data storage and processing capabilities for DNA sequencing. Bloom filters (BFs) are such techniques used in bioinformatics domain for classification where a set of known DNA sequences facilitate the classification of the unknown sequences. It is due to the inherent capability of BFs that allows it to answer any query in either yes or no. Suppose a bloom filter is created for a set of elements and the set is used to answer the query if a certain element is present in the set or not. Further, to determine if the queried element is part of the set, m hash functions are applied to that element. The index positions obtained through hashing are checked in the BF. If all those indexes (or bits) of BF are set to 1, this means that the element is most probably part of the set (false positives can arise in BF), else the element is certainly not in the set. The query response time is fast as it takes O(m), where m is the number of hash functions applied to the element. Likewise, a BF created for a known DNA sequence can determine if the given unknown sequence has any similarity with the known DNA sequence (for which BF is created) and this can quickly be answered in a series of yes or no. Thus, BFs highly assist in determining the origin of the given unknown sequence. In addition to their use as classifiers, some work exists on compression side as well. However, there are certain problems that limit the use of this fast and memory efficient data structure in other areas of bioinformatics.

As mentioned earlier, a bloom filter can only answer any query in yes or no. For example, if there exists a BF for a human chromosome 16 and if a certain disease pattern needs to be checked whether it is present in that chromosome or not, the BF can answer the query in “yes” if the pattern is present or “no” if the pattern is not present. However, in medical science, it is significant to determine the presence of the pattern in the certain chromosome but also the position of that pattern in the chromosome. It is because the location of the disease pattern can also determine the type of the disease; e.g., Maturity Onset Diabetes of the Young (MODY), which is an inherited form of diabetes mellitus, has more than ten different types. Based on the location of the pattern, the type of the disease can be found. Moreover, the number of times a certain pattern is reported in the DNA sequence is also important as it shows the intensity of the disease. For example, the high repetition of the pattern CTG/CAG is often linked with the Huntington's disease. However, algorithms have been previously proposed where compression is achieved through BFs but to retrieve the original sequence it must be decompressed. Through this paper, we addressed some of these problems due to which BFs are not particularly preferred for pattern matching and sequence compression. Following are the main contributions of this work:

- We propose an algorithm using multiple BFs to compress DNA sequences which eliminates the need to store the original DNA sequence for pattern searching tasks
- We propose and integrate techniques for identifying the exact location of the pattern as well as the number of times the pattern is repeated in the sequence, within the compressed sequence
- We provide initial experimental results for evaluating the accuracy and time efficiency of the proposed approach along with deficiencies observed and identify potential future research directions

The remainder of this paper is organized as follows: Section 2 covers the related work. The proposed algorithm is discussed in Section 3.  Section 4 presents the experimental results and finally the last section presents the conclusions.

## 2. Literature Review

Compression algorithms for DNA or genome generally fall into four categories. The first category is naive-bit encoding where one or more characters are represented by a certain codeword. For example, the simplest encoding for DNA sequence can be obtained by assigning 2 unique pair of bits to each of the unique alphabet present in a DNA sequence like A=00, C=01, G=10, and T=11 [1–3]. The second category is a dictionary-based or substitutional compression. It is mostly observed that a DNA sequence consists of a lot of repeated sequences. As a result, the repeated sequences can be replaced by references to a dictionary that is either built offline or is maintained at runtime [4]. The most common dictionary-based algorithms include LZ77 and LZ78.

Further, there are statistical methods that achieve extremely good compression rates by generating probabilistic models based on genome datasets [5–7]. The fourth category is referential compression where any repeated sequence in an input dataset is replaced with a reference to one or more external DNA sequences [8–11]. A data structure plays a critical role in any algorithm designed for achieving good compression ratios, fast searching of patterns inside sequences, or both. Many algorithms proposed in the area of bioinformatics make use of self-index-based data structures to achieve above mention goals. They can be used to avoid the need of keeping large files of text along with the index [12–15]. The index itself contains sufficient information that any part of the text can be recreated. Some of the examples of self-indexes include compressed suffix array (CSA), Succinct Suffix Array (SSA), and FM-index [16–19]. In addition to this, there are other variations of indexes as well [20] and some of them are shown under different categories in Table 1.

Bloom filter (BF), a probabilistic data structure, is often seen being used in different algorithms. In [21], BF is used to store the DeBruijn graph created for storing genome; otherwise DeBruijn itself consumes lot of memory to store the data. However, this leads to false nodes and branching due to false positives induced by BF but the authors have proposed a mechanism to reduce them. Rozov et al. [22] proposed BARCODE that makes use of reference sequence as mentioned previously to achieve compression. The bloom filters are core part of their algorithm; they hash all the reads in the BF and decode them later for querying. Stranneheim et al. [23] proposed FACS that uses BF to classify sequences and the classification speed is high as the searching time is independent of the BF size. In [24], Sequence Bloom Tree (SBT) is proposed that also makes use of BF for fast classification of sequences.

Similarly, in bioinformatics, DNA error correction is another interesting problem where blood filter is used. Due to large-sized data sets, error correction is a time-consuming process. Therefore, a number of algorithms have been proposed for error correction. A. Ramachandran et al. [25] have proposed an FPGA based accelerated error-correction algorithm. It is designed to improve the throughput of DNA. The proposed algorithm is based on BLESS which used bloom filter as a main data structure due to its memory efficiency.

The genomic process generates a huge amount of data which is often difficult to store. Therefore, many techniques have been proposed to store the data in compressed form. In [26], Sebastian proposed a compression algorithm to process single and paired reads. The proposed technique is based on partial matching and dynamic Markov coder algorithm. However, the main drawback of the proposed work is slow processing and large memory usage. The slow process is due to the query searching process typically for incomplete k-mers. Similarly, in [27], the authors proposed a new mechanism to generate an index of similar strings. The proposed technique can also manage redundancies. Tomasz et al. [28] proposed a new technique to store and index the NGS reads in memory. The proposed technique is based on the traditional technique of counting and locating k-mers. The proposed technique is more compact compared to GkA and CGkA solutions.

Heo. Y. et al. [29] have proposed bloom filter based error correction for Next Generation Sequencing (NGS) data. With tremendous increase in the size of data, the NGS data contains far more errors compared to traditional sequencing methods. The traditional algorithms are not memory efficient. Therefore, the authors proposed an algorithm based on bloom filters to reduce the memory overhead and produce much more accurate results. Bloom filters are widely used for pattern matching; however, there are many applications where bloom filters are not suitable, e.g., deleting one attribute based on the other. Jiangbo Qian et al. [30] proposed bloom filter based associative deletion algorithms. Reem Khairy et al. [31] presented the accelerated bloom filter approach using high level synthesis. The authors presented the portable solution that can be incorporated in many applications to take benefit of bloom filter performance. In bioinformatics, most of the algorithms proposed using bloom filter belong to the category of classification. The BF can easily determine if a sequence belongs to the text being searched or not but it cannot accurately determine the exact location of the pattern and the number of times the pattern is repeated in the sequence. These are important factors to be determined if the exact disease or intensity of the disease is to be identified as discussed in the previous section. The next section covers the proposed approach which is based on Multiple Bloom Filters to identify the location of pattern in a given sequence.

Similarly, an efficient storing collection of large populations of genomes and timely searching of data is a challenging problem. Techniques exist to reduce the space requirement such as reference-based compression. However, such storing techniques adversely impact the search techniques. Therefore, Sebastian et al. proposed a framework based on Multi-Reference Compressed Search Indexes (MRCSI) [32]. The proposed MRCSI has achieved increased compression rates and support string searching. In genomes, aligning sequencing reads is one of the key operations performed against a large collection of genome data. The aligning process required a significant amount of memory. In [33] Danek et al. proposed a Multiple Genome Index (MuGI) to find the occurrences in a large collection of genomes. The MuGI used small size customizable indexes and easily runs on commodity systems with 8 GB RAM.

The next section covers the multiple bloom filter based proposed approach for storing chromosome data and searching patterns.

## 3. Multiple Bloom Filters (MBFs) Based Proposed Approach

**Figure 1:**
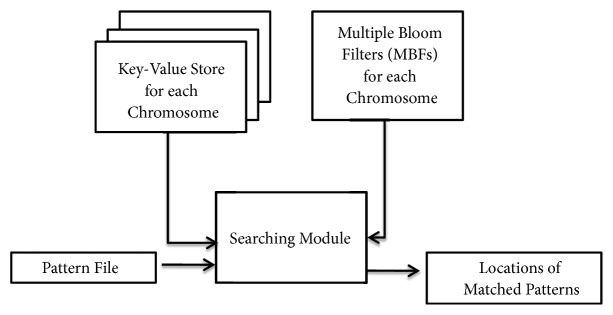
Overview of proposed approach.

This section presents the proposed method of storing chromosome data and searching patterns in the data as shown in Figure 1. First, we describe the method of using MBFs or an array of BFs to store chromosome data in the compressed format. Subsequently, we describe the procedure to search patterns in the DNA sequences that are stored in MBFs without decompressing them.

### 3.1. Compression

This phase comprises of three important steps:

1. Formation of k-mers of the given chromosome
2. Storing locations of each unique k-mer in the Key-Value (KV) store on disk
3. Storing location data for each unique k-mer of chromosome in a separate BF

The first step is critical to determine the length (k) of DNA words where a DNA word is simply a sequence of characters (A,C,G,T) of length k. If the length of DNA word “k” is chosen as 4, 5, or 6, the associated words of a given DNA sequence is referred to as 4-mers, 5-mers, and 6-mers, respectively. If the length of the sequence (N) is segmented into words is already known, then the number of total words formed can easily be determined based on the selected k-mer size (k), for example,

  given sequence: ACGTTCCACGTTCA,
  total characters: N=14,
  k-mer size k=5,
  total no. of words= N-k+1 =14-5+1= 10.

**Figure 2:**
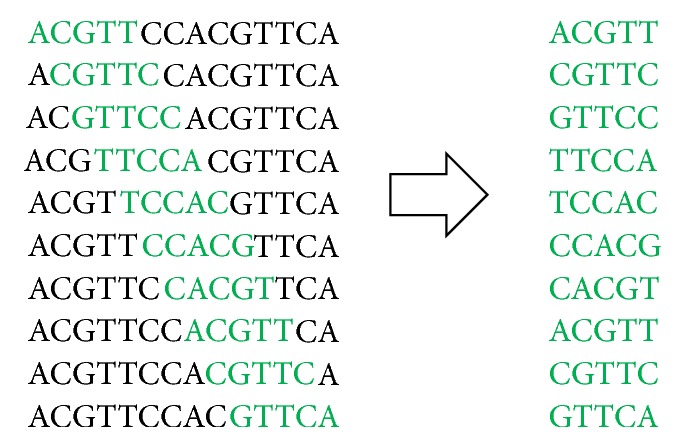
Formation of words.

**Figure 3:**
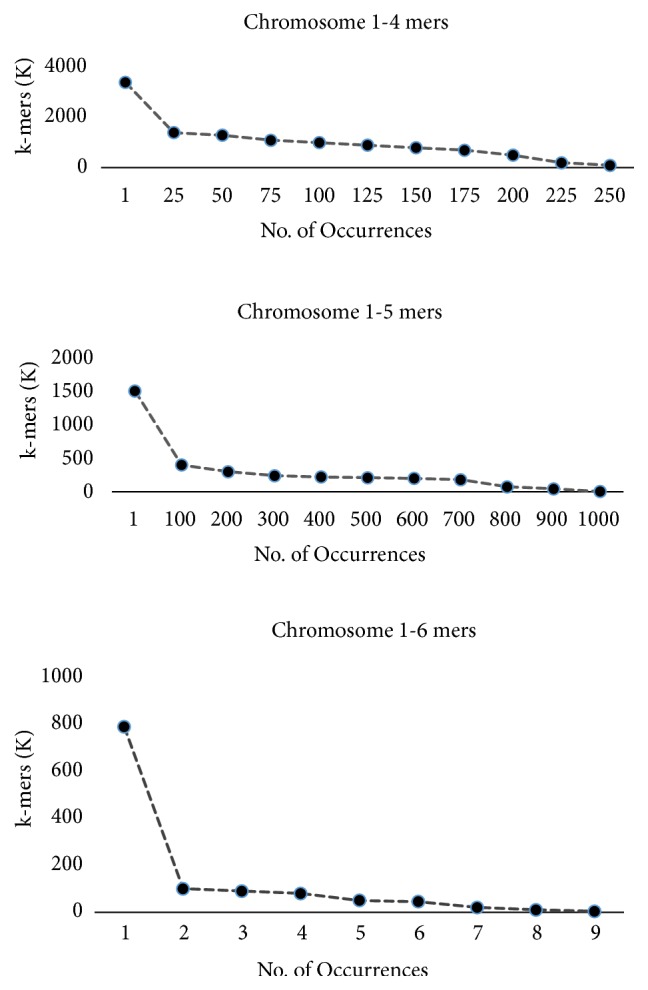
Shows the number of occurrences of k-mers in chromosomes.

Figure 2 shows the formation of words for the above sequence. As we increase k-mer size, the number of unique k-mers increases by the factor 4k. When k is increased, the number of unique k-mers or words increases; on the other hand k-mer occurrences in the sequence reduce. We carried an experiment for different chromosomes; Figure 3 shows this trend particularly for chromosome 1 (Chr 1). Instead of displaying each k-mer with characters (e.g., AAAA, AAAC, and AAAG for k=4) on x-axis, we have just shown their numbers like k-mer 1, k-mer 2, and k-mer 3. The y-axis shows the number of occurrences of each particular k-mer in the sequence. It is an important observation that increasing k-mer size increases number of unique k-mers whereas the frequency of occurrence of the k-mers gets reduced in the sequence as length of the DNA words is increased. Thus, k-mer size is a critical parameter and has different impact on the size of KV store and MBF. This is further discussed in the experimental results section.

Next, a Key-Value (KV) storage is required to store all the locations of each unique k-mer in the given chromosome. Here, Key is the k-mer (or DNA word), e.g., ACGTT, and Value is the sequence of locations where that specific k-mer is located in the chromosome.  Table 2 shows how location data is saved in KV store corresponding to each unique k-mer and it resides on the disk. The left side of the table maintains k-mers and right side stores the indexes or locations of each corresponding k-mer in the sequence that needs to be compressed. This step is performed only once and in offline mode before pattern matching process starts. It has special significance in this proposed solution as it is used to answer a single query per pattern. This is further discussed in the pattern matching section.

**Figure 4:**
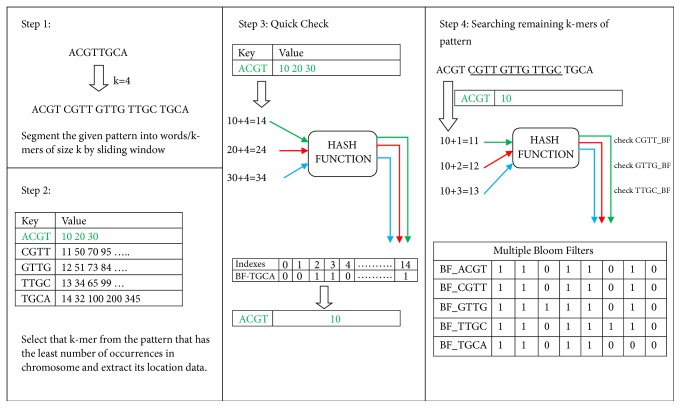
Illustration of usage of MBFs.

The next step is the construction of Multiple Bloom Filters (MBFs) for each single chromosome. The MBFs are an integral part of the proposed scheme. Bloom filter (BF), a probabilistic data structure, is used here to store all the location data of each unique k-mer in a separate BF. As a result, there exists a BF for each distinctive k-mer present in the chromosome. This consequently leads to the formation of MBFs as shown in Figure 4. A BF is just like a vector that stores Boolean values: 0 and 1. By default, all the values are set to false (0) and to store the locations of the specified k-mer in BF, first hash function is applied to the location (or index value) of the k-mer and the resultant value is used to find that index of the BF, which is then set to true (1). Thus, there exists a BF for each unique k-mer and the BF has all of its indexes set to true that are found after applying a hash function to each location (or index) where that k-mer is present in the chromosome.

The size of the BF plays a significant role in achieving compression. To determine the accurate size of the BF, following parameters should be known: n= number of items to be stored in the BF:

p= probability of false positives (FPs);
m= number of bits in the filter;
k= number of hash functions (used for index finding in BF).
  (1) m=nlnpln2
  (2) k=mnln2

Here, false positives refer to the situation where a k-mer is not present at a particular location in the chromosome whereas the hash function results in indexes that are set to true in BF, which means k-mer is present at that location. For instance, we need to store one of the locations (which is a 20th index of chromosome string) of k-mer TTTT, a hash function is applied on 20, and the result is an index 10 of BF corresponding to TTTT. Index 10 of BF is then set to true. Further, a query needs to be answered if TTTT is present at index 30 of the chromosome string or not, a hash function is applied to it (i.e., 30), and the result comes out to be index 10, which is earlier set to true. Results show that TTTT is present at index 30 of the chromosome string whereas BF is set to true at index 10 due to 20. In this way, false positives can create a discrepancy in results. The false positive can be reduced if “p”, i.e., FP, is kept low and large number of hash functions (i.e., “k”) is used in (1) and (2), respectively. In the proposed solution, we kept FP probability neither too high nor too low, i.e., 0.01 but n varies as each k-mer has a different number of occurrences in the chromosome. Consequently, m that is the size of the bloom filter in bits varies for each unique k-mer of the specified chromosome.

### 3.2. Pattern Searching

A pattern is a sequence of DNA characters (A,C,G,T) that is to be searched in a DNA sequence or chromosome. Before the pattern matching process starts, a pattern is decomposed into k-mers of size k. DNA word or k-mer size is set to 4 when KV store is built as discussed in the previous section. The 4 is selected as the final value of k and a pattern is decomposed into (N-k+1) k-mers where N is the length of the pattern sequence. Afterwards, one k-mer that is part of the pattern is selected such that it has the least number of occurrences in the chromosome (target string to be searched) among all other k-mers that are part of a pattern. For example, a KV store and MBFs are created for a DNA sequence. Now, a pattern ACGTTGCA is to be searched inside that sequence. As k is already set to 4 when KV store is built, therefore 8-4+1=5 words of size 4 each is formed for the pattern, which is as follows:

  ACGT CGTT GTTG TTGC TGCA

The next step is to select the k-mer from the pattern with the least number of occurrences in the sequence. In case, there is a tie between two or more k-mers then the one that comes first in the sorted list of k-mers is selected. Suppose ACGT is one such k-mer in the present example with only three occurrences in the sequence. Then, a query is sent to KV store to provide the location data of ACGT that comprises of all the index positions of DNA sequence where ACGT has occurred (indexes 10, 30, and 40). Afterwards, this data has to be reduced to make pattern matching process faster. For that quick check step is performed, that requires the bloom filter of the last or the first k-mer of the pattern depending on the position of the k-mer chosen in the previous step.

In the given example, BF of TGCA, which is the last k-mer of pattern, is extracted. Since the distance between ACGT and TGCA is 4; therefore, for each location index of ACGT present in the location data extracted from KV store, 4 is added, e.g., 10+4, 30+4, and 40+4. The resultant value (i.e., 14 first) is hashed and the result of the hash function (index) is checked in the BF of TGCA if it is set to 1 or not. If it is set to 1 then there is a high possibility that the given pattern exists in the chromosome; otherwise pattern does not exist. For each location of ACGT for which the BF of TGCA gives back a positive response, the location is preserved and for the ones that give a negative response, those locations are discarded from the temporary array. In this way, this array often gets reduced in size as the pattern matching process proceeds as illustrated in Figure 4 as well. The usefulness of this step is highlighted when patterns are longer and it can also be observed in the experimental section that large length patterns give many accurate results compared to smaller patterns.

Now, the 10th index is the only index left in the temporary location array after quick check process. So, this index, i.e., 10, is incremented by 1 and the resultant index 11 is hashed. The result of the hash is used to find out if that place in the bloom filter of CGTT (which is the second k-mer of the pattern) is set to 1 or 0. Since it is set to 1, this means ACGTT (ACGT and CGTT) is present in the sequence. Next, the same procedure is performed for GTTG and TTGC with location indexes 12 and 13, respectively. In the given example, BFs of both GTTG and TTGC give positive results. However, if any BF returns a negative response in the form of 0 then pattern matching process does not need to proceed further as the pattern does not exist. By the end of this pattern matching process, the temporary array of ACGT is left with only those indexes where the pattern exists.

## 4. Experimental Results

First, an experiment is performed to determine the impact of k-mer size on Key-Value (KV) store. As mentioned earlier, KV store is an integral part of the proposed solution, which stores all the locations of each unique k-mer present in the chromosome. We used LevelDB [34] for this purpose, which is a fast on-disk KV store that stores sorted data on the basis of the key in the compressed form (“LevelDB”, 2011).  Table 3 shows the size of KV store for Chr 1, Chr 12, and Chr 21 for the different k-mer sizes (4, 5, and 6). The chromosome datasets are obtained from NCBI and all the “N” characters are removed from the datasets before conducting the experiments on them [35].

The results show that, by incrementing the k-mer size by one, the size of the KV store increases drastically. Therefore, we chose k-mer of size 4 for the main experiments that are discussed in this section. The KV store can be created offline and stored on disk before the start of pattern matching process as a result construction time is not discussed here. Further, some experiments are performed to see the impact of k-mer size on the size of Multiple Bloom Filters (MBFs) for each corresponding chromosome. The false positive probability of each of the bloom filter is set to 0.01. In Table 4, it is observed that incrementing the k-mer size keeps the MBF size nearly constant for each corresponding chromosome. For example, MBF size of Chr 1 is 268 MB, 270 MB, and 271 MB for k-mer sizes 4, 5, and 6, respectively. Moreover, construction of MBF for chromosomes is fast and is nearly the same for a certain chromosome irrespective of its k-mer size. The false positive (FP) probability of a bloom filter plays a significant role in achieving compression; see Table 5. We determined the size of MBF for Chr 1, Chr 12, and Chr 21 on three different FP probabilities: 0.1, 0.01, and 0.001. It is observed that high FP probability achieves good compression as compared to low FP probability. However, choosing high FP probability can have a negative impact on pattern matching results whereas the increase in pattern size can reduce the effect on results as discussed later in this section.

Next, we evaluated the performance of the proposed solution by measuring the time it takes to find the pattern inside the chromosome. The results are listed in Table 6, where the first column represents the pattern used for evaluation, followed by the length of the pattern and afterward the time taken to identify the stated number of occurrences of that pattern inside the string of the specific chromosome. In all these experiments, k-mer size is kept 4 and FP probability is 0.01. If we increase FP probability, the MBFs get highly compressed; however at the same time the number of false positives in the pattern matching process increases that leads to increase in pattern matching time. The number of false positives for Chr 1, Chr 12, and Chr 21 is reported in Table 7 for two cases. In the first case, FP probability is set to 0.1 and for the second case, it is 0.01. It shows that increasing FP probability drastically increases the number of false positives; on the other hand, as we increase the length of the pattern, the number of FPs gets highly reduced as shown for the pattern TTTATTGGAAATATGGGAT present in Chr 12. Thus, this problem can be resolved with large length of patterns (20-25) used for identification. In addition to this, a restriction can be placed on the number of occurrences of the pattern to be identified. We compared the results with another pattern matching tool named Seeq [36] that is based on a Levenshtein distance metric. The results are shown in Table 8. It is observed that MBF based pattern matching is fast but its speed is not comparable with other pattern matching tools. Thus, some optimizations are required to make this pattern matching process faster.

## 5. Conclusion

Problems related to storage and processing are not unknown in the field of bioinformatics due to the large volume of DNA sequence data. In this paper, bloom filters are explored to provide a solution other than the simple classification of DNA sequences. The proposed approach using MBFs allows finding the exact location of the patterns and the number of times the pattern is repeated in the compressed sequence, without using original DNA sequence in its decompressed form. The experiments are carried out on human chromosomes to provide a proof of concept application of the proposed MBFs technique in real life data. Optimizations on various levels are still required and in future, we aim to work on all such areas that lack efficiency, e.g., KV store size and search time of patterns. We expect that this solution can bring highly promising results if optimizations are taken in the right direction. The ability to look for patterns inside compressed DNA sequences makes the proposed solution feasible for real-world applications and is anticipated to be highly sought after in the near future.

## Figures and Tables

**Table 1:** Classification of data structures.

Data Structure	Variants
Full-Text Index	Suffix Array [20]
Full-Text Index	Suffix Tree [20]
Self-Index	Compressed Suffix Array (CSA) [16]
Self-Index	Run Length Compressed Suffix Array (RLCSA) [12]
Self-Index	Succinct Suffix Array [13]
Self-Index	FM-Index [17]
Self-Index	Alphabet Friendly FM-Index [18]
Self-Index	LZ-Index [19]
Word-based Self-Index	Word based Compressed Suffix Array (WCSA) [14]
Word-based Self-Index	Word based Succinct suffix array (WSSA) [14]
Word-based Self-Index	Byte oriented Codes wavelet Tree (BOC-WT) [37]
Probabilistic-Index	Fast and Accurate Classification of Sequences (FACS) [23]
Probabilistic-Index	Probabilistic de Bruijn Graph [21]
Probabilistic-Index	Bloom filter Alignment-free reference-based Compression and Decompression (BARCIDE) [22]
Probabilistic-Index	Sequence Bloom Tree [24]

**Table 2:** Key-Value store.

Key	Value (K-mer positions)	
ACGTT	0	7

CACGT	6	
CCACG	5	

CGTTC	1	8

GTTCA	9	
GTTCC	2	
TCCAC	4	
TTCCA	3	

**Table 3:** Impact of k-mer size on Key-Value store.

K-mer size	Chr 1	Chr 12	Chr 21
	KV Store Size (GB)	KV Store Size (MB)	KV Store Size (MB)
4	1.2	700	195.1
5	1.3	756.8	209.8
6	1.4	816.4	226.4

**Table 4:** Impact of k-mer size on MBFs and construction time (false positive probability of bloom filters= 0.01).

K-mer Size	Chr 1		Chr 12		Chr 21	
	MBF Size (MB)	Construction Time (Secs)	MBF Size (MB)	Construction Time (Secs)	MBF Size (MB)	Construction Time (Secs)
4	268	25.4	153	14.68	41.5	4.21
5	270	30.9	154.8	16.29	41.9	4.5
6	271.5	33.2	155.4	17.49	42.04	5.01

**Table 5:** Impact of false positive probability on MBF size (k-mer size=4).

Chromosome		FP Prob=0.1	FP Prob=0.01	FP Prob=0.001
	Original file size (MB)	MBF Size (MB)	MBF size (MB)	MBF size (MB)
Chromosome 1	230.8	134.5	268	402.2
Chromosome 12	131.9	76.9	153	230.6
Chromosome 21	35.9	20.81	41.5	62.24

**Table 6:** Pattern searching time (k-mer size=4; FP probability =0.01).

Pattern	Length	Chr 1		Chr 12		Chr 21	
		Occurrences	Time (Secs)	Occurrences	Time (Secs)	Occurrences	Time (Secs)
TTTGAT	6	98196	137.10	59222	36.27	16169	2.31
CATCAT	6	77840	83.81	46203	25.41	12202	1.85
GTGTCTGT	8	8615	88.87	5035	23.70	1471	1.50
TGGAATGGGA	10	552	116.64	286	28.70	108	1.85
TTTTTTAGAAT	11	327	81.48	208	23.14	60	1.48
GAGGCAGGAGGATCCC	16	82	32.14	44	9.28	13	0.65
TTTATTGGAAATATGGGAT	19	0	34.37	1	9.93	0	0.66
AGCATATTTTTACTGTAGGAGAA	23	0	34.08	1	9.97	0	0.66

**Table 7:** Number of false positives for FP probability=0.1 and 0.01.

Pattern	Length	Chr 1		Chr 12		Chr 21	
		FB Prob=0.1	FB Prob=0.01	FB Prob=0.1	FB Prob=0.01	FB Prob=0.1	FB Prob=0.01
TTTGAT	6	39260	9172	23743	5666	6438	1549
CATCAT	6	39567	9906	23097	6007	6453	1620
GTGTCTGT	8	7309	1251	3982	756	1207	206
TGGAATGGGA	10	636	104	339	49	55	14
TTTTTTAGAAT	11	431	75	261	50	69	9
GAGGCAGGAGGATCCC	16	36	18	29	10	3	0
TTTATTGGAAATATGGGAT	19	0	0	1	1	0	0
AGCATATTTTTACTGTAGGAGAA	23	0	0	1	1	0	0

**Table 8:** Pattern searching time in seeq.

Pattern	Length	Chr 1		Chr 12		Chr 21	
		Occurrences	Time (Secs)	Occurrences	Time (Secs)	Occurrences	Time (Secs)
TTTGAT	6	98196	1.624	59222	0.932	16169	0.258
CATCAT	6	77840	1.606	46203	0.902	12202	0.275
GTGTCTGT	8	8615	1.49	5035	0.856	1471	0.261
TGGAATGGGA	10	552	1.341	286	0.807	108	0.22
TTTTTTAGAAT	11	327	1.36	208	0.79	60	0.215
GAGGCAGGAGGATCCC	16	82	1.224	44	0.721	13	0.204

## Data Availability

No data is associated with this article to report.

## Appendix

The code and dataset used to benchmark the proposed technique is available at https://github.com/mrhua2019/Pattern-Matching-for-DNA-Sequencing-Data-using-Multiple-Bloom-Filters.git.
